# Depressive Symptoms among Fourth Form Students in St. Kitts and Nevis High Schools

**DOI:** 10.1100/tsw.2009.16

**Published:** 2009-02-28

**Authors:** Gillian A. Lowe, Garth Lipps, Sharon Halliday, Amrie Morris, Nelson Clarke, Rosemarie N. Wilson

**Affiliations:** ^1^ Department of Community Health and Psychiatry, University of the West Indies, Mona, Jamaica; ^2^ Department of Sociology, Psychology and Social Work, University of the West Indies, Mona, Jamaica; ^3^ Ministry of Health and the Environment, Government of St. Kitts and Nevis, Nassau, Bahamas; ^4^ Ministry of Health, Government of St. Vincent and the Grenadines, Nassau, Bahamas; ^5^ School of Clinical Medicine and Research, University of the West Indies, Nassau, Bahamas

**Keywords:** prevalence, depressive symptoms, St Kitts & Nevis, adolescents

## Abstract

There has been limited research on depressive symptoms among high school students in St. Kitts and Nevis. This project examines levels of depressive symptoms among fourth form (grade 10) students attending all high schools in St. Kitts and Nevis. Students enrolled in the fourth form during the 2006/2007 academic year in all high schools were administered the Beck Depression Inventory II (BDI-II). A near census of the students was conducted (n = 744 students; 50.4% females, 47.6% males, and 2% no gender reported; age 13–19 years, mean = 15.5 ± 0.8 years). Six in every ten students (62.1%) reported some symptoms of depression, with 14.8% reporting moderate to severe and 9.7% reporting severe symptoms of depression. Females reported significantly higher BDI-II scores (t_(727)_ = 7.11, *p* < 0.01) with 70% of females reporting some level of depressive symptoms compared with 52% of their male counterparts (^X^2^^(1) = 24.6, *p* < 0.05). Additionally, 34% of females were in the moderate to severe or severe range of depressive symptoms, while 15% of males were in the same range. Students who were older than expected for their grade (i.e., 17 years or older) reported significantly higher BDI-II scores (F(2,740) = 2.88, *p* < 0.05) than students who were younger or at the expected age (i.e., 14–16 years). Students whose mothers had a high school or postsecondary education reported significantly lower levels of depressive symptoms than students whose mothers had less than a high school education (F(3, 637), = 4.23, *p* < 0.05). Symptoms of depression among fourth form students in St. Kitts and Nevis are a prevalent problem that is influenced by students’ age, gender, and social class as indicated by maternal education.

### BACKGROUND

Limited research has been conducted on adolescent depression among students in the Caribbean[1,2,3]. Even less research has been conducted on teenage emotional health in countries such as St. Kitts and Nevis. The present study will contribute to the body of literature on Caribbean adolescent mental health by examining the characteristics and prevalence of depressive symptomatology among fourth form (grade 10) students in St. Kitts and Nevis. Results of this investigation may provide information that will aid the school system in St. Kitts and Nevis to be more attentive to students' emotional health.

#### Depression among Adolescents

Depression is the most common psychiatric disorder of adolescence[4]. Its consequences include unplanned pregnancy, academic and social derailment, and, most seriously, attempted and completed suicide[4]. Longitudinal studies of adolescents with depression have shown high rates of recurrence with progression to chronicity and conversion into adult affective disorders[4]. Additionally, epidemiological studies suggest that there may be a greater risk for depressive symptoms among females that first emerge around the period of mid-adolescence and increase with students' age[5,6]. Past regional and international studies with nonclinical adolescent samples have reported prevalence rates of 2–9%[2,7,8,9,10].

Much of the research on adolescent depression has been done with other Caribbean as well as North American and European samples (e.g.; [2,3,6,11,12,13,14,15]). Relatively little research on depression has been conducted using youth from St. Kitts and Nevis. Searches of PubMed, Sage Online, and PsychInfo did not reveal any studies that examined depression in adolescents in St. Kitts and Nevis.

While the past research is valuable, the society of St. Kitts and Nevis is distinct from that of North America. In contrast to the populations of the U.S. and Canada (Table 1), the vast majority of the population of St. Kitts and Nevis are of African descent or of mixed race (97%)[16,17,18], with the majority of residents living in rural parts of the islands (51.1%)[16,17,18]. Residents of St. Kitts and Nevis also tend to be poorer[19]. Nearly half of all households in St. Kitts and Nevis are headed by single women (47%)[20,21,22]. Finally, the population of St. Kitts and Nevis has fewer people with postsecondary education (2.1%)[16,17,18], with the majority of residents having a secondary level of education (67.2%)[16,17,18].

#### Background of High School Students in St. Kitts and Nevis

Provision for secondary school education was started in 1968[23]. The country presently offers universal access to secondary education, where every student from the primary school system is guaranteed a place in the secondary educational sector[23]. Unlike other Caribbean territories, e.g., Jamaica, St. Kitts and Nevis has no exit examination for students leaving the primary schools and entering high schools[23,24]. Instead, students feed from primary schools into high schools to which they are assigned[24].

In accordance with past research using non–West Indian samples[25,26,27], it is hypothesized that:

1. Female adolescents will report more and stronger symptoms of depression than their male counterparts despite male students' underachievement.
2. Depression scores will increase with students' age.

**TABLE 1**
**Demographic and Social Features of St. Kitts and Nevis, the U.S., and Canada**

| | St. Kitts | U.S. | Canada |
|---|---|---|---|
| Population[a] | 48,000 | 304,486,000 | 33,304,000 |
| Percentage rural[b] | 51.1% | 24.8% | 23.4% |
| Ethnicity | | | |
| African descent[b,c] | 97%[b] | 12%[b] | 3%[c] |
| European[b] | 2% | 76% | 86% |
| Gross national income per person[a] | 10,430 | 45,850 | 35,310 |
| Postsecondary education[b] | 2.1% | 39.8% | 44.4% |
| Single female–headed households | ≈ 47%[d] | 12.5%[e] | 12.7%[f] |

[a] PRB (Population Reference Bureau) World Population Data Sheet.

[b] http://www.atlapedia.com/online/countries/stkitts.htm
http://www.atlapedia.com/online/countries/unitedst.htm
http://www.atlapedia.com/online/countries/canada.htm

[c] Statistics Canada. Ethnic Origin and Visible Minorities, 2006. Census Catalogue No.: 97-562-X2006008

[d] United Nations Committee on the Elimination of All Forms of Discrimination Against Women. Summary Record of the 563rd Meeting. 2002. CEDAW/C/SR.563.

[e] U.S. Census Bureau, 2005–2007. American Community Survey America's Families and Living Arrangements: 2006.

[f] Statistics Canada. Families and Households, 2006 Census of Population. Statistics Canada Catalogue No. 97-553-XCB2006.

3. Students' social class as indicated by parental education will be associated with depressive symptoms such that students whose mothers have higher levels of education[1] will be found to have lower depression scores on the BDI-II.
4. Age and gender will combine to accelerate age-related increases in depression among female adolescent students.

## METHODS

### Sample

A near census of fourth form students attending all high schools in Saint Christopher (St. Kitts) and Nevis was conducted (n = 744). A list of all schools providing secondary education in St. Kitts and Nevis was obtained from the Ministry of Education. Schools were visited and all fourth form students in attendance on that day were surveyed. The students who were surveyed closely matched published statistics on the enrolment students in secondary schools in St. Kitts and Nevis. Estimates from the UNESCO Institute for Statistics[28] for the 2006/2007 school year suggest that the population of fourth form students should be approximately 800 students with an equal distribution of male and female students. In line with these estimates, 50.4% of our students were female and 47.6% were male, with 2% not reporting their gender.

[1] We used maternal education as an indicator of students' social class as a large number of St. Kitts and Nevis families are headed by women. As such, the use of maternal education as an indicator of students' social class facilitates the operationalization of social class.

Students in the fourth form should be between 14 and 16 years of age. Students in our sample ranged from 13 to 19 years of age (mean = 15.5 $\pm$ 0.8 years).

### Measures

#### *Beck Depression Inventory - II (BDI-II)*

The BDI-II[29] is a 21-item questionnaire that examines the cognitive, behavioral, affective, and somatic symptoms of depression. Each item of the BDI-II is comprised of a series of rank-ordered statements. Each statement is assigned a score from 0 to 3, reflecting the severity of the symptom. Students were asked to circle the number associated with the statement that most accurately describes their feelings during the past 2 weeks. Previous research suggests that the BDI-II is reliable in North American samples of adults[29]. Studies using both nonclinical[6,30] and clinical[31] samples of adolescents have reported acceptably large internal consistency reliabilities, with coefficient alphas ranging from 0.87 to 0.94. Within a sample of Jamaican adolescents, the BDI-II had a reasonable degree of internal consistency reliability ($\alpha$ = 0.87)[1]. Past research suggests that the BDI-II has validity across different cultures[14,29], even cultures that place a high stigma on psychological problems[32].

#### *Demographic Data*

A variety of information on students' demographic features, including their age and gender, was collected using a series of brief questions modeled after those used in the Census of Population[33].

### Procedure

Prior to the start of the project, one of the authors liaised with the Ministry of Education and the schools to secure their participation in the project. All schools were surveyed over the period of a single week. Data for the project were collected from students during one of their regularly scheduled classes. All fourth form students who were present on the day their school was surveyed were given a package of instruments to complete. This package consisted of an informed consent form for the adolescents, the BDI-II, a series of questions regarding their demographics, as well as other research instruments.

### Statistical Analyses

A variety of statistical analyses were conducted. Preliminary analyses were conducted to check and correct data capture problems using simple frequency and cross-tabulations. A check was made on the extent of missing values for each question on the questionnaire. No question was found to be missing more than 10% of responses. On this basis, the mean score was substituted for missing values on each question of the BDI-II. Basic descriptive statistics were then run to check that the data met the statistical assumptions required by chi-square analysis and t-tests. These checks found that the data met the assumptions needed to perform these tests. As such, no transformations were made to the data. Simple frequency distributions were conducted to estimate the prevalence of depressive symptoms in fourth form students.

### RESULTS

Six in every ten students (62.1%) reported some symptoms of depression, with 14.8% reporting moderate to severe and 9.7% reporting severe symptoms of depression.

#### Gender

Females reported significantly higher BDI-II scores (Table 2; $t_{(727)} = 7.11$, $p < 0.01$), with 70% of females reporting some level of depressive symptoms compared with 52% of their male counterparts ($\chi^2(1) = 24.6$, $p < 0.05$). Additionally, 34% of females were in the moderate to severe or severe range of depressive symptoms, while 15% of males were in the same range.

**TABLE 2**
**BDI-II Mean Scores by Key Demographic Variables**

| | Mean | Median | Range |
|---|---|---|---|
| Gender | | | |
| Females | 17.1 | 16.0 | 0–58 |
| Males | 11.7 | 10.0 | 0–60 |
| Age | | | |
| 14 | 15.0 | 17.0 | 0–48 |
| 15 | 14.0 | 11.0 | 0–60 |
| 16 | 14.2 | 12.0 | 0–55 |
| 17 | 17.6 | 17.0 | 0–46 |
| Maternal education | | | |
| Primary | 14.5 | 12.0 | 0–47 |
| Secondary | 15.4 | 13.0 | 0–60 |
| Associate/vocational | 12.8 | 10.0 | 0–44 |
| University | 12.7 | 9.5 | 0–43 |

#### Age Effects

ANOVA, examining the relationship of age to BDI-II depression scores, indicated that there was a significant main effect of age on depression scores (Table 2; $F(3, 740) = 2.88$, $p < 0.05$). However, contrary to our hypothesis, we did not find a linear increase in depression scores as age increased. Instead, there appeared to be U-shaped relationship between age and depression scores such that students who were younger or older than expected for their grade reported higher depression scores. Post hoc contrasts of this effect found that students who were older than the expected for their grade reported significantly greater depression scores.

#### Age and Gender Effects

To explore the interactive relationship of age and gender on students' depression scores, a two (Gender) by four (Age) ANOVA analysis was conducted. Contrary to hypothesis 4 above, we did not find a statistically significant interaction of age and gender on BDI-II depression scores ($F(3, 721) = 0.95$, $p >$

0.05). Instead, separate statistically significant main effects were found for age (F(3, 721) = 3.48, $p <$ 0.05) and for gender (F(1, 721) = 37.87, $p < 0.05$).

#### Social Class

To examine social class differences in depression, we utilized the mother's highest level of education as an indicator of social class. St. Kitts and Nevis has the highest percentage of female-headed households in the Caribbean[34]. Nearly half (43–47%) of all households in St. Kitts and Nevis are headed by a single female[19]. Given this fact, we used the mother's highest level of education as an indicator of social class rather than the father's education. To facilitate these analyses, we grouped mothers' level of education into four categories: less than secondary, high school, vocational training and associate degree, and bachelors and graduate degrees. Maternal education was significantly associated with students' BDI-II scores (Table 2; F(3, 637), = 4.23 , $p < 0.05$). Students whose mothers had either vocational training and associate degrees, or bachelors and graduate degrees, reported lower mean BDI-II scores than students whose mothers had a secondary education only.

### DISCUSSION

Nearly a quarter (24.5%) of all students showed significant symptoms of depression. In keeping with our hypotheses, female students reported significantly higher BDI-II scores than males. Contrary to our hypotheses, the relationships between age and depression, maternal education and depression were more complex. Students who were either older or younger than expected for their grade reported higher BDI-II scores than those who were on track. Similarly, students whose mothers had some postsecondary education reported significantly lower symptoms of depression than those whose mothers had a secondary education. Students whose mothers had a primary school or lower level of education did not report significantly higher or lower BDI-II scores than those whose mothers had postsecondary education.. We did not find support for our hypothesis that age and gender would interact to increase BDI-II depression scores.

Compared to international studies of the prevalence of depressive symptoms, students in St. Kitts and Nevis appear to report higher mean BDI-II scores (14.5 vs. 7.2), with substantially higher percentages of students falling into Shaw et al.'s[35] suggestions for moderate and severe cut-points for BDI depression scores (41.5 vs. 13.1%)[36]. This difference in the distribution of depression scores may be accounted for by ethnicity and social conditions. Previous Caribbean research on adolescent depression[2] found higher mean BDI-II scores in Caribbean adolescents than in adolescents in North America[6,14].

St. Kitts and Nevis has a number of social issues that may contribute to higher levels of depressive symptoms among adolescents. These include the family structure, whereby many adolescents grow up in female-headed single-parent families with higher levels of poverty and social inequality[19,34]. Growing up in a single-parent home may produce greater financial stress and a reduction of family support[37]. Students in St. Kitts and Nevis must deal with limited opportunities for personal advancement. Students who leave at the fourth form or graduate from high school have the options of working, receiving trade-vocation education, going abroad to attend university and pay their living expenses, or paying high tuition fees to foreign-based universities at home.

Our finding of a gender difference in depressive symptoms is consistent with previous international research that found a 3:1 ratio of depressive symptoms in favor of males[38,39]. In this study, we found that there was a 2:1 ratio of moderate to severe symptoms of depression in favor of male students.

Often students are older than expected for their grade as they have previously failed a grade or have been held back for another reason. Similarly, students who have skipped a grade may be younger than their peers. Past research has linked grade retention to higher levels of depression[40]. Additionally, Thompson et al.[41] found that the relative age effect was linked to students' emotional adjustment such that students who were young for their grade reported higher negative self-evaluation than those whose age was appropriate for their grade level. Consistent with the international research cited above, we found that students who were off-track reported higher BDI-II scores than their on-track counterparts.

Parental education has been used in the Caribbean as an indicator of social class[2,10,39]. In the present study, we used maternal education to represent the social class of children's families. Previous research in the U.K. has shown that social class is a strong moderator in the production of depressive symptoms[42]. It is possible that the greater opportunities provided by better education provides greater human, material, and financial resources for children. This greater support may play a protective role in preventing symptoms of depression.

Previous research in the Caribbean has linked maternal education to depressive symptoms among offspring[2,10,39]. Students whose mothers had secondary education or less reported higher symptoms of depression than those whose mothers some postsecondary education. It is possible that mothers who have postsecondary education may have a more optimistic view of themselves and society, and as such may be more encouraging and facilitating of their children's growth and development. In contrast, mothers who have not progressed to postsecondary education may be frustrated with their opportunities for growth and development, and communicate a more pessimistic view of life and opportunities for advancement to their children. This difference in perspective may explain the differences in symptoms of depression reported by children[43].

#### Limitations

Several factors limit the relationships reported in this study. First, the project examined depressive symptomatology as assessed by the BDI-II and not clinical depression. As such, the prevalence of depression may be higher or lower than we report. Second, given the shared historical and cultural forces, we assume that the BDI-II is equally valid and reliable among St. Kitts and Nevis adolescents as it is among Jamaican students. Third, due to the cross-sectional nature of the study, we cannot infer a causal relationship between the variables studied and depressive symptoms. Fourth, it is possible that the selection of fourth form students may have artificially increased the prevalence of depressive symptomatology in this project due to the fact that low-performing fourth form students may be denied the opportunity to continue their education. Schools in St. Kitts and Nevis require that students performing below average are not permitted to continue on to the fifth form and, therefore, university and college education. Finally, the current study only examined depressive symptoms among fourth form students. As such, our findings may not generalize to all adolescents attending high schools in St. Kitts and Nevis.

### CONCLUSIONS

Symptoms of depression among fourth form students are a prevalent problem that is influenced by students' age, gender, and social class as indicated by maternal education.
